# Detection of ESKAPE Bacterial Pathogens at the Point of Care Using Isothermal DNA-Based Assays in a Portable Degas-Actuated Microfluidic Diagnostic Assay Platform

**DOI:** 10.1128/AEM.02449-16

**Published:** 2017-02-01

**Authors:** Lars D. Renner, Jindong Zan, Linda I. Hu, Manuel Martinez, Pedro J. Resto, Adam C. Siegel, Clint Torres, Sara B. Hall, Tom R. Slezak, Tuan H. Nguyen, Douglas B. Weibel

**Affiliations:** a Department of Biochemistry, University of Wisconsin-Madison, Madison, Wisconsin, USA; b Department of Biomedical Engineering, University of Wisconsin-Madison, Madison, Wisconsin, USA; c Department of Chemistry, University of Wisconsin-Madison, Madison, Wisconsin, USA; d Leibniz Institute of Polymer Research, Dresden, Germany; e Department of Mechanical Engineering University of Puerto Rico at Mayagüez, Mayagüez, Puerto Rico, USA; f Computations Directorate, Lawrence Livermore National Laboratory, Livermore, California, USA; g Physical and Life Sciences Directorate, Lawrence Livermore National Laboratory, Livermore, California, USA; h Global Security Principal Directorate, Lawrence Livermore National Laboratory, Livermore, California, USA; Chinese Academy of Sciences

**Keywords:** ESKAPE pathogens, bacteria, detection, infection, isothermal PCR, lab-on-a-chip, point of care

## Abstract

An estimated 1.5 billion microbial infections occur globally each year and result in ∼4.6 million deaths. A technology gap associated with commercially available diagnostic tests in remote and underdeveloped regions prevents timely pathogen identification for effective antibiotic chemotherapies for infected patients. The result is a trial-and-error approach that is limited in effectiveness, increases risk for patients while contributing to antimicrobial drug resistance, and reduces the lifetime of antibiotics. This paper addresses this important diagnostic technology gap by describing a low-cost, portable, rapid, and easy-to-use microfluidic cartridge-based system for detecting the ESKAPE (Enterococcus faecium, Staphylococcus aureus, Klebsiella pneumoniae, Acinetobacter baumannii, Pseudomonas aeruginosa, and Enterobacter spp.) bacterial pathogens that are most commonly associated with antibiotic resistance. The point-of-care molecular diagnostic system consists of a vacuum-degassed microfluidic cartridge preloaded with lyophilized recombinase polymerase amplification (RPA) assays and a small portable battery-powered electronic incubator/reader. The isothermal RPA assays detect the targeted ESKAPE pathogens with high sensitivity (e.g., a limit of detection of ∼10 nucleic acid molecules) that is comparable to that of current PCR-based assays, and they offer advantages in power consumption, engineering, and robustness, which are three critical elements required for the point-of-care setting.

**IMPORTANCE** This paper describes a portable system for rapidly identifying bacteria in resource-limited environments; we highlight the capabilities of the technology by detecting different pathogens within the ESKAPE collection, which cause nosocomial infections. The system is designed around isothermal DNA-based assays housed within an autonomous plastic cartridge that are designed with the end user in mind, who may have limited technological training. Displaying excellent sensitivity and specificity, the assay systems that we demonstrate may enable future diagnoses of bacterial infection to guide the development of effective chemotherapies and may have a role in areas beyond health where rapid detection is valuable, including in industrial processing and manufacturing, food security, agriculture, and water quality testing.

## INTRODUCTION

The discovery and clinical introduction of penicillin in the 1940s is arguably one of the most important scientific achievements in the history of medicine. Unfortunately, penicillin-resistant Staphylococcus aureus strains were identified in hospitals very soon after the widespread use of penicillins (1–3). Antibiotic resistance is a serious threat to human health and a significant challenge for modern medicine. In the United States, 11,285 deaths per year are attributed to methicillin-resistant Staphylococcus aureus (MRSA), making it more prevalent than Parkinson's disease, emphysema, homicides, and HIV/AIDS combined (4, 5). To highlight the magnitude of infectious diseases in the area of human health and the potential scale of drug-resistant bacterial infections, an estimated 106 million new global cases of gonorrhea occurred worldwide in 2008, and 3.1 million lower respiratory infections and 1.5 million diarrheal diseases accounted for two of the top 10 global causes of death in 2012 (6). Enterococcus faecium, S. aureus, Klebsiella pneumoniae, Acinetobacter baumannii, Pseudomonas aeruginosa, and Enterobacter spp. comprise the ESKAPE panel of bacteria and cause the majority of hospital-related infections in the United States (7). These organisms consistently “escape” the effects of many clinical antibiotics and are a growing threat to public health (8).

The timely and accurate identification of the causative agent responsible for an infection is the critical first step in effective patient care (9, 10). For bacterial infections, this first step guides patient treatment strategies and effective usage of antibiotics. Proper drug use is necessary to mitigate the growing emergence of antibiotic resistance (11). The current gold standard for bacterial identification is based on the isolation and growth of strains and their identification using established phenotypic screens, such as determining patterns of growth under different nutrient conditions (12). However, closely related bacterial species and different strains may share similar metabolic profiles, and growth-based assays are slow (from hours to days). The requirements of sterile lab conditions, expensive capital equipment (greater than $100,000), and microbiology-trained personnel place these techniques out of reach for users in regions that lack these resources, including many areas of the developing world.

Nucleic acid amplification and molecular diagnostic techniques, such as PCR, are displacing laboratory culture-based methods of bacterial identification. The use of PCR-based diagnostic tools in the point-of-care setting has been hampered because of thermocycling requirements with PCR assays. Isothermal techniques for nucleic acid amplification and detection have circumvented key technical and resource limitations of PCR-based assays and make them feasible at the point of care (13, 14). Recombinase polymerase amplification (RPA) is an isothermal method that uses a recombinase enzyme to direct the binding of primers to template DNA and amplify a target DNA sequence in a strand displacement format (15). RPAs can detect as few as ∼10 copies of target DNA and operate at a low constant temperature (∼42°C) that minimizes power consumption and simplifies heating and power handling. RPAs have been used to detect bacterial, viral, and eukaryotic human pathogens, including Francisella tularensis (16), Plasmodium falciparum (17), Mycobacterium tuberculosis (18), yellow fever virus (19), and HIV (20). Currently, there are several barriers to bringing RPAs to the point of care, including reagent costs associated with large-scale operations, few available mechanisms that simplify the steps for manipulating reagents and performing assays (21), and the limited repertoire of small electronic components for quantifying assays, communicating results, and making recommendations on therapies.

This paper describes a solution to bridge the technology gap and bring diagnostic tests to end users at the point of care who make therapeutic decisions on antibacterial chemotherapies. We developed a low-cost, portable, rapid, and simplified field assay for detecting ESKAPE bacteria that we refer to as “B-chip” (for bacterial detection chip). B-chip is a degas-driven microfluidic cartridge that performs dozens of multiplexed RPAs to detect ESKAPE bacterial pathogens from a small sample volume (22, 23). B-chip consists of a disposable plastic assay cartridge for detecting ESKAPE organisms using degas-driven fluid flow to simplify liquid handling steps and minimize contamination that interfaces with an integrated incubation-detection system consisting of an inexpensive heat control and optical system housed within a three-dimensional (3D)-printed enclosure. We envision that B-chip will impact the detection of human and agricultural pathogens in a range of geographic areas, including rural areas where resources are limited.

## RESULTS AND DISCUSSION

### Primer and probe development.

We developed unique probe and primer sets for detecting E. faecium, S. aureus, K. pneumoniae, A. baumannii, and P. aeruginosa; we were unable to successfully develop probe and primer sets for Enterobacter. Our choice of RPA was based on its tolerance for amplifying DNA in unpurified samples containing bacteria, the simplicity of designing primers, and its low operating temperature, which would reduce the power requirement of the electronic B-chip reader and maximize its battery life.

We first performed a multiple-sequence alignment of each of the 5 input genomes to locate the most evolutionarily stable elements of the target organism. By focusing our oligonucleotide design on evolutionarily stable regions of DNA, we increased the selectivity of assays against strains of target organisms for which genomes are not currently available, and we increased the detection of future strains and variants of each target bacterium. In the second step, we performed a sequence search against all known bacterial, viral, and protist genomes, as well as against a small representative set of fungal genomes; the data size and computational complexity limited the number we could compare. We ruled out regions of DNA in target organisms for RPA oligonucleotides if there was similarity to nontarget sequences. These steps enabled us to locate regions of DNA in the target genomes that are both conserved and unique.

We used the software Primer3 for primer and probe oligonucleotides (24, 25). Adjusting the oligonucleotide sequence parameters to fit the design guidelines for RPA enabled us to generate candidate sequences for each target. We scored each combination of candidate sequences based on its profile of melting temperature, oligonucleotide hybridization stability, and potential for dimerization. Self-hybridization is a more significant problem for RPA primers than for other assays based on DNA amplification (e.g., reverse transcription-PCR [RT-PCR]) because the tetrahydrofuran spacer contributes to several more stable (i.e., nonproductive) oligonucleotide conformations than the existing probe. Consequently, we performed another step of screening for self-hybridization. We used the UnaFold software (26) to generate probability dot plots and self-hybridization likelihoods of potential oligonucleotide conformations. We selected oligonucleotide sequences with a low self-affinity under predicted reaction conditions and chose primer and probe combinations with the best scores for a target locus as the candidates for testing in DNA amplification assays.

We initially developed 8 primer-probe sets for each of the 5 organisms and performed a BLAST search of all primer-probe set sequences against sequence information available for all species within these genera on GenBank using the PriMux software (27). This step enabled us to determine that the assays we were developing amplified only targeted genetic material and did not produce a false-positive result when challenged with nontarget DNA. We performed RPA reactions on all 8 primer-probe sets in the absence of the exonuclease, in which we added purified target genomic DNA from 2 to 6 strains (from ATCC, Table S1) of bacteria within each species. This step created an intact DNA amplicon that we separated on a 4% agarose gel to verify that the assays produced the predicted product size. Using RPA reaction mixtures containing the exonuclease, we determined a detection time for primer-probe sets that yielded the correct product size with all of the strains of target genomic DNA. Using rapid detection time as a criterion, we downselected assays further and moved the two best-performing RPA primer-probe sets to testing on a B-chip. The selected probe-primer combinations and the targeted genomic regions are summarized in Table S3.

### B-chip design and operation.

**FIG 1:**
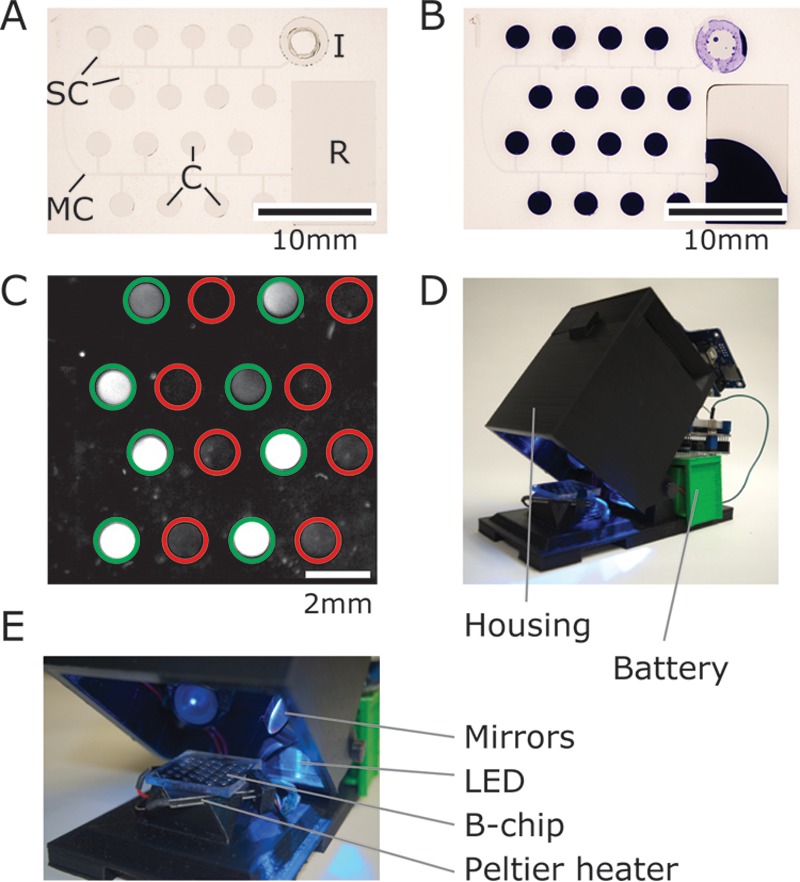
Images depicting the B-chip and electronic reader. (A) An image of a B-chip containing 16 microchambers (C, each with a volume of 1 μl). (B) An image of a degassed B-chip that has been loaded with an aqueous solution of crystal violet (50 μl) at the inlet “I” after 20 min. Air fills the main and side channels (MC and SC, respectively), physically separates reactions, and prevents cross-contamination between the chambers. Excess sample is collected in the reservoir “R” (scale bar, 10 mm). (C) Representative image of fluorescence from on-chip RPAs for S. aureus, imaged with an ImageQuant; green circles indicate the presence of S. aureus-specific primers, and red circles indicate the absence of primers (scale bar, 2 mm). (D and E) Images of the B-chip reader highlighting the housing, optical components, and a Peltier plate to heat the sample.

For this study, we designed B-chips in poly(dimethylsiloxane) (PDMS) consisting of a single long channel connected by short channels to 16 microchambers that each had a volume of 1 μl (Fig. 1A). The design consisted of one layer of PDMS (∼5-mm thick) containing the embossed channels and chambers and a flat layer of PDMS (∼5-mm thick) that are conformally sealed together to create the fluidic features. Before sealing, we pipetted a 1-μl droplet containing primers, fluorescent exoprobe (a disordered DNA sequence containing a fluorescent dye and quencher that binds to an amplified DNA sequence), and RPA enzymes into each chamber and evaporated the liquid. These B-chips were designed to accommodate the analysis of a 50-μl sample. After removing chips from a vacuum, ∼15 min was required to fill/load the device using a 50-μl sample volume (Fig. 1B); the fill time can be reduced by altering the design and physical dimensions (28). We chose the 50-μl sample volume to ensure that after each microchamber was filled completely and that the reagents were dissolved (16 μl total volume for all of the microchambers), excess sample was pumped into the reservoir, and a plug of air was pulled through the inlet and filled the central channel to isolate liquid samples in each microchamber and avoid cross-contamination by diffusion (22). We found it important to add the magnesium acetate (MgOAc) for the RPA during the sample loading step (typically we added it to the sample), as adding it to the reagents and drying it down in the chambers produced a high level of nonspecific fluorescence, including in chambers lacking primers (data not shown).

After incubating for 30 min at 37°C, we imaged the fluorescence of the microchambers and used image analysis scripts to quantify probe fluorescence and indicate amplification (Fig. 1C). We also found that B-chips can be incubated for >60 min or at higher temperatures compatible with loop-mediated isothermal amplification (LAMP) assays (∼60°C) without any detectable loss of liquid in the chambers due to evaporation.

### Characterizing isothermal DNA amplification on B-chips.

**FIG 2:**
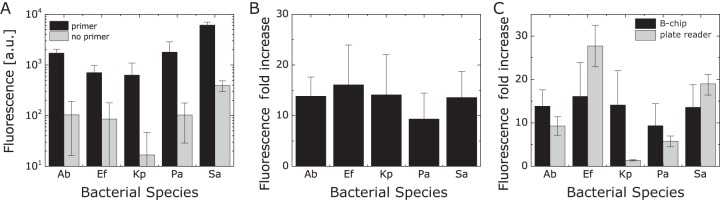
Quantifying RPA fluorescence in B-chip and plate reader assays. (A) A plot depicting the fluorescence intensity of RPAs to test isolated DNA from the following bacteria: A. baumannii (Ab), E. faecium (Ef), K. pneumoniae (Kp), P. aeruginosa (Pa), and S. aureus (Sa). We tested RPAs in the presence and absence of primers. (B) A plot depicting the fluorescence fold increase, which is a comparison of the RPA microchamber fluorescence in the presence of primers to the absence of primers for strains tested in panel A. (C) Comparison of the fluorescence fold increases between B-chip and the plate reader assay (*n* = 3).

We used two criteria for selecting the optimized primers/probes: (i) the intensity of the fluorescence readout, and (ii) the ratio of fluorescence intensity of positive sample (with primers) compared to background fluorescence (no primers) (Fig. 2A). We detected a 10- to 15-fold increase in the fluorescence intensity of B-chip assays for each of the five organisms we tested when samples consisted of genomic DNA isolated from a single bacterial strain (Fig. 2B); the fluorescence fold increase corresponds to the fluorescence ratio between microchambers containing primers and those without primers (Fig. S2). Our observed values are consistent with previously reported RPA fluorescence values for shrimp white spot syndrome virus (29). Using this assay to guide our selection, we selected one probe-primer pair per species to study the sensitivity and specificity of our RPAs.

To benchmark our B-chip RPAs, we compared on-chip fluorescence values using the optimized primer-probe pairs with values from the same assay performed on a plate reader in a larger volume (50 μl). The fluorescence fold increases we observed between the two assays were largely comparable (Fig. 2C), indicating the suitability of the microfluidic detection device compared to more sensitive techniques. Standard curves for the fluorescent reporter 6-carboxyfluorescein (FAM) suggest that plate reader and B-chip readings are comparable (Fig. S3). For unknown reasons, the primer-probe set for K. pneumoniae consistently performs poorly in microplate wells yet works well on-chip.

### Sensitivity of probe-primer pairs.

**FIG 3:**
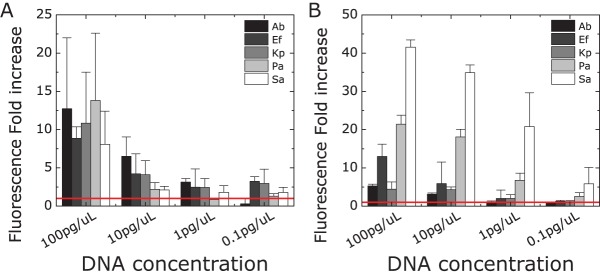
Determining the sensitivity of probe-primer pairs in on-chip RPAs. Determination of the fluorescence fold increase of RPAs containing primer versus no primers in B-chip (A) and plate reader (B) assays. We analyzed the sensitivity of each probe-primer set against different concentrations of isolated genomic DNA (0.1, 1, 10, and 100 pg/μl). The red line indicates no amplification (no-primer control) (*n* = 3). A. baumannii, Ab; E. faecium, Ef; K. pneumoniae, Kp; P. aeruginosa, Pa; S. aureus, Sa.

We analyzed the sensitivity of the primers and probes using purified genomic DNA samples isolated from each species (Table S1). We measured DNA concentrations from 0.1 to 100 pg/μl, which correspond to approximately 10 and 10,000 copies of DNA per microchamber, respectively. We compared B-chip and plate assay readouts (Fig. 3). In general, the plate assay is slightly more sensitive than the B-chip assay, which may be due to the differences in reaction volumes. In both formats, the fluorescence fold difference increased with increasing DNA concentration, suggesting the possibility of performing quantitative detection of DNA titers with on-chip and plate-based RPAs.

When quantifying the slope of the fold increase in fluorescence versus DNA concentration, we found plate reader assays [slope = 0.11 (in picograms per microliter)^−1^] to be slightly more sensitive than the B-chip [slope = 0.097 (in picograms per microliter)^−1^] (Fig. S4). The plate reader detected as few as 10 copies of DNA with high reliability, consistent with reported values for RPAs (29), corresponding to 100 to 1,000 copies of DNA on-chip (based on the molecular weight of the target genomic DNA). The sensitivity of detection also varied with each bacterial DNA species, which suggests that the quality of the primer-probe pair strongly affects the fluorescence readout. We detected concentrations of 10 pg/μl for A. baumannii and E. faecium, 1 pg/μl for K. pneumoniae and P. aeruginosa, and as low as 0.1 pg/μl for S. aureus, which corresponds to ∼10 copies of genomic DNA per μl. This level of sensitivity may enable the detection of subclinical cases of bacterial infections, which can prove important in other applications, e.g., surveillance of the spread of pathogens in livestock. We used a gold-standard method of DNA extraction (i.e., commercially available DNA extraction kits; see Materials and Methods) in all of our experiments, which made it possible for us to keep this variable constant and focus our development on optimizing the assay, B-chip cartridge, and B-chip reader. Although we are not familiar with a commercial product that has miniaturized this DNA extraction method to work in resource-limited environments, we view this step as important for system development and planned in future stages, when testing samples in remote environments, to incorporate one of the many exciting technologies developed for point-of-care DNA isolation (30, 31) and develop a fully integrated point-of-care device.

### Specificity of probe-primer pairs.

To determine the specificity of the optimized probe and primer pairs, we analyzed whether probe-primer pairs detected their corresponding target bacterial DNA without binding DNA from the other ESKAPE strains. We prepared samples to test each probe-primer pair against DNA samples of each ESKAPE species. The primers and probes did not display cross-reactivity in a plate reader RPA (Fig. S5). We found that the measured fold fluorescence intensity increased for nonspecific combinations of primer-probe-DNA at the zero-amplification level (Fig. S5, red line). We observed significant amplification levels only in the presence of the corresponding primer-probe-specific target DNA. We did not observe any false-positive/true-negative signals for all the tested species. These results indicate the level of specificity of the selected primer and probes, which represented a key factor for a building an effective bacterial detection device.

**FIG 4:**
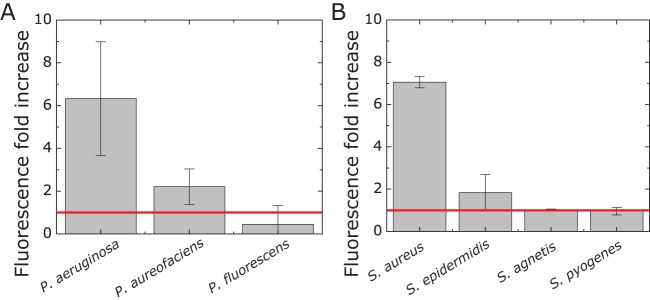
Testing the specificity toward different related strains. Plots depicting the fluorescence fold increase for RPAs of selected ESKAPE species toward closely related strains of Pseudomonas spp. and Staphylococcus species. The red line indicates no amplification (no-primer control) (*n* = 3).

The observed specificities of the primers and probes are unsurprising, as the ESKAPE bacteria are genetically distant. We tested the specificity of the probe-primer pairs for selected ESKAPE species toward more closely related strains from the same genus/species (compare Fig. S6 and Table S2). As a starting point, we chose a few species from the genus Pseudomonas and a selection of clinical isolates of Staphylococcus species (Rodney Welch, UW-Madison hospital) which have been used previously in our lab (23), to evaluate species specificity. We observed that the probe-primer pairs for P. aeruginosa and S. aureus were specific for the selected species they were designed to detect. These probe-primer pairs did not detect the other tested Pseudomonas or Staphylococcus species (Fig. 4). To detect P. aeruginosa cells, we grew a cell culture to an absorbance (λ = 600 nm) of ∼0.6, diluted the culture to 10^−6^ to 10^−8^, and lysed the culture with a mixture consisting of 200 μg/ml lysozyme, 1.5 mM EDTA, and 1% SDS. After incubation for 20 min, we loaded the lysed samples without further purification onto the B-chip and ran an RPA on-chip. We were able to detect P. aeruginosa DNA in all samples with a fluorescence fold increase comparable to that for pure P. aeruginosa DNA (Fig. S7). This methodology indicates the potential of combining the B-chip with simple DNA extraction solutions for the analysis of bacterial samples, which we are currently developing.

### Selectivity of primer-probe sets.

**FIG 5:**
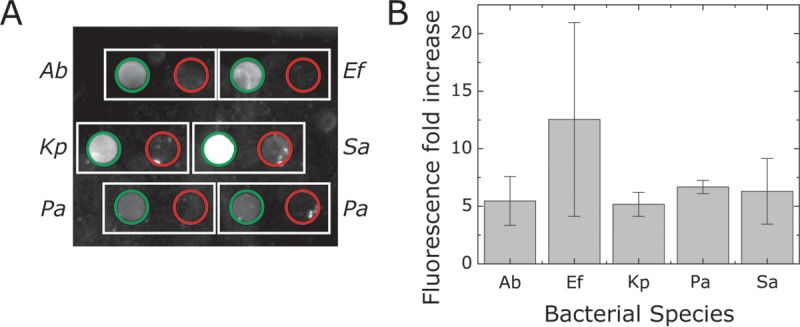
Determining the selectivity of primer-probe pairs in RPAs of samples containing DNA mixtures. (A) A plot of RPA fluorescence in microchambers containing bacterium-specific primers (green circles) or no primers (red circles) using samples containing a mixture of all 5 bacterial genomic DNAs at a concentration of 100 pg/μl. (B) A plot depicting the fold increase of fluorescence levels (ratio) between primer and no primer in samples consisting of mixtures of equal amounts of genomic DNA of all five organisms (*n* = 3). A. baumannii, Ab; E. faecium, Ef; K. pneumoniae, Kp; P. aeruginosa, Pa; S. aureus, Sa.

To determine assay selectivity on-chip, we tested the primers and probes against a mixed solution containing five of the six ESKAPE genomic DNAs (Fig. 5A). We compared the fluorescence intensities to the single genomic DNA detection experiment and found a slight decrease in the fluorescence fold increase with samples containing mixtures of DNA (Fig. 5B, 5- to 12-fold) compared to the individual DNA samples (Fig. 2B, 10- to 15-fold increase). In the presence of nonspecific DNA, the B-chip assays succeeded in detecting target ESKAPE strains, indicating the potential applicability for the chip in relevant point-of-care situations.

### Time-dependent detection profile of DNA in B-chip assays.

**FIG 6:**
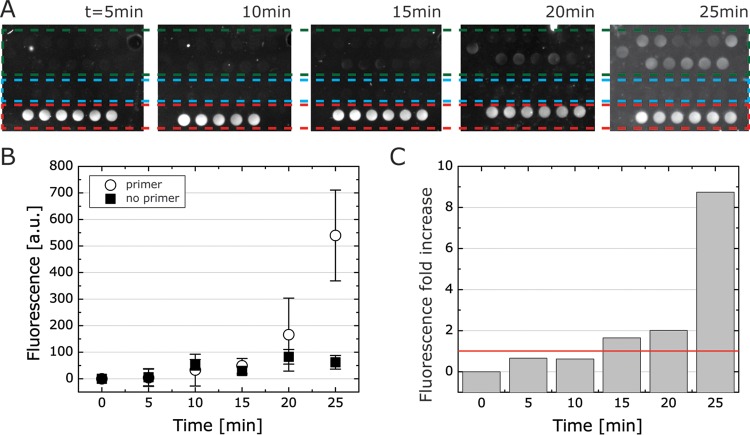
A time lapse of B-chip RPAs for S. aureus detection using an ImageQuant. (A) A panel of images depicting the RPA fluorescence readout from on-chip assays of S. aureus with primers (green dashed box), no primers (blue dashed box), and 6-FAM control (200 nM, red dashed box); we quantified fluorescence using an ImageQuant. (B) Fluorescence over time for primer versus no primer. a.u., arbitrary units. (C) Fluorescence fold change for data from panel B, and red line indicates fold change at 1.

RPA is a fast isothermal amplification technique, and the assay signal typically saturates within 15 to 30 min at 39 to 42°C. To evaluate the time for detection on-chip, we tested RPAs incorporating S. aureus probe/primers for detecting pure S. aureus DNA samples over a 25-min time interval (Fig. 6A). We measured the fluorescence intensity of RPAs with primers compared to assays lacking primers and compared the fluorescence intensity to a positive control consisting of 6-FAM (Fig. 6B). Within 20 to 25 min, the fluorescence intensity reached a 9-fold increase in fluorescence (Fig. 6C), suggesting that on-chip RPAs can detect samples within 25 min, which is considerably faster than a standard PCR assay (45 to 60 min). RPA is an isothermal reaction that does not require temperature cycling, thereby reducing its operating time compared to PCR-based detection.

### Development and characterization of the portable electronic reader.

We designed a portable B-chip detection system for measuring assay fluorescence containing an excitation source, camera, incubation heater, fluorescence filters, and software to control the individual components. All of the components are open source and available online. We designed the cartridge pedestal to accommodate chips that are square, ∼6.5 cm^2^ in surface area, and several millimeters thick. We used four light-emitting diodes (LEDs) for fluorophore excitation, in which each LED was aimed at one side of the B-chip and oriented so that it was 45° with respect to an axis normal to the PDMS top surface (i.e., aimed at the surface of the chip at an oblique angle). Each LED had a 135° viewing angle (full width at half maximum [FWHM]). Orienting the LEDs at a 45° angle enabled light from each LED to impact two faces of the PDMS side walls, thereby scattering light more efficiently inside the PDMS and the microchambers.

**FIG 7:**
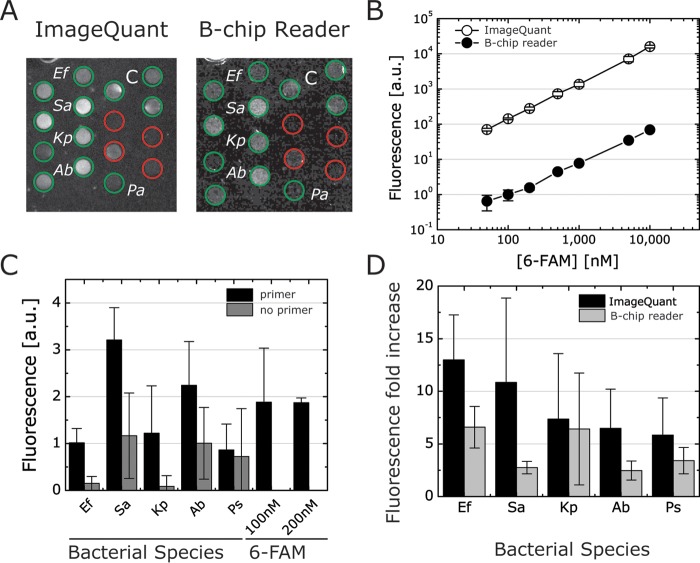
Comparison of B-chip RPA fluorescence detection using an ImageQuant and an electronic reader. (A) Examples of the fluorescent readout of all ESKAPE genomic DNA samples in B-chip microchambers (500 μm tall) imaged with an ImageQuant and a B-chip reader (green circles represent primers are present; red circles indicate no primers; C indicates a control). (B) Standard curve of 6-FAM fluorescence in B-chip microchambers analyzed with ImageQuant and B-chip reader (see also Fig. S7, Supplemental Information). (C) Fluorescence readout of ESKAPE collection with the B-chip reader (100 and 200 nM indicate control fluorescence intensities of the 6-FAM dye). (D) Comparison of fluorescence fold increase (ImageQuant versus B-chip reader) of primer versus no primer for the ESKAPE collection (*n* = 3). A. baumannii, Ab; E. faecium, Ef; K. pneumoniae, Kp; P. aeruginosa, Pa; S. aureus, Sa.

We used the complementary metal-oxide semiconductor (CMOS) camera to measure various concentrations of 6-FAM in chambers on-chip to benchmark fluorescence detection and compare it to the fluorescence of 6-FAM we anticipated would be produced by RPAs (Fig. 7). Using the ImageQuant LAS 4000, we found that the typical positive RPA result closely correlated with a concentration of 100 nM 6-FAM probe dissolved in buffer or water (the exoprobe concentration is 120 nM, according to the kit instructions). We tested the detection range of the CMOS camera with 6-FAM concentrations varying from 50 nM to 10 μM and containing 14 mM MgOAc. We observed a linear relationship between fluorescence intensity and 6-FAM concentration (Fig. 7B). The low fluorescence intensity we measured with 100 nM 6-FAM with the B-chip reader led us to set the microchamber height to 500 μm to increase the optical path length for detection. We optimized several parameters of the reader to enable the CMOS to detect low light levels from assays, including the power supplied to LEDs, the type of emission filters, the amount of ambient light entering the chamber, the amount of LED light refracting from the PDMS device edges, and the height of the chambers.

We analyzed the performance of the B-chip detection system and compared the fluorescence to measurements performed with an ImageQuant (Fig. S8, Supplemental Information). Degassed B-chips containing 6-FAM were loaded with a solution of 14 mM MgOAc to yield microchambers containing 6-FAM concentrations ranging from 50 nM to 10 μM. The fluorescence measurements produced by the reader are less sensitive than the ImageQuant, as the benchtop system captures 16-bit images rather than the 8-bit captured by the CMOS. We found the fluorescence intensities were linear for the relevant concentration range of reagents used in the assay (Fig. 7B).

To explore and demonstrate the capabilities of the reader for field applications, we performed test runs for the ESKAPE collection using the device interface and heating system and compared the final result to the ImageQuant measurements (Fig. 7A). We used the experimental setup and B-chip assay preparation described above. We preloaded chambers on B-chips with primers of ESKAPE organisms and RPA enzymes and then dried, assembled, and degassed them. To run assays, we filled B-chips with samples of genomic DNA for each ESKAPE species dissolved in a solution of MgOAc and incubated them for 30 min in the B-chip electronic reader using the internal heating system. The B-chip reader results reproduced our previous RPA results (e.g., see Fig. 5) with fluorescence intensities comparable to those of the 6-FAM controls (Fig. 7C). Not surprisingly, we found that the fluorescence fold increases were lower for the B-chip reader than for the ImageQuant due to the reduced detection sensitivity (Fig. 7D). Optimizing the optical train and electronic control elements in future versions of the reader will improve its sensitivity.

### Conclusion.

This paper describes the combination of a simple and portable cartridge-based RPA and a versatile electronic reader that can be used to perform assays in the field. Using degas-driven cartridges provides a unique platform for packaging RPAs that can simplify their use and potentially reduce contamination by packaging and compartmentalization. We successfully identified 5 of the ESKAPE strains using on-chip RPAs with a portable B-chip reader that has detection limits that are generally comparable to those of more expensive large-scale laboratory equipment used for the detection of bacterial pathogens. A challenge with these assays in their current form is that they are unable to differentiate between live and dead bacteria, which may lead to false-positive results. One way to transcend this limitation is to shift to detecting mRNA (rather than DNA) by taking advantage of its higher rate of degradation once released from cells into the environment. We are currently exploring this approach.

The absence of temperature cycling associated with isothermal RPAs substantially reduces (by 2 times) their time compared to standard PCR-based assays. Our results indicate that the sensitivity of on-chip detection will enable us to detect subclinical concentrations of bacteria in a range of other infections, including in asymptomatic bacteriuria, that have a titer of bacteria that matches the sensitivity of the B-chip (32). The versatility of the design, potential low cost of the system, and ease of use make the B-chip platform applicable to bacterial detection at the point of care. We envision that the B-chip will improve diagnostics in clinical and agricultural settings by providing health experts with rapid and accurate information on the microbial composition of samples. The result may improve the accuracy of antibiotic therapies, or in some cases, avoid the use of antibiotics altogether, and support management efforts aimed at reducing the emergence of antibiotic-resistant bacteria and their transmission.

## MATERIALS AND METHODS

### Bacterial strain growth conditions and DNA preparation.

We designed RPAs for detecting E. faecium, S. aureus, K. pneumoniae, A. baumannii, and P. aeruginosa. All strains used in this study were grown in lysogeny broth (LB) medium at 37°C with shaking at 200 rpm, with the exception of E. faecium, which was grown in brain heart infusion (BHI) broth. For initial tests, we purchased purified DNA for each species from the American Type Culture Collection (ATCC) (information about the DNA samples is summarized in Table S1). To test the specificities of our designed primers and probes, we assayed purified genomic DNA from several related bacterial strains for each species; we isolated DNA from overnight cultures of bacterial strains using the UltraClean microbial DNA isolation kit (Mo Bio Laboratories, Carlsbad, CA) and quantified DNA concentrations using a NanoDrop 1000 spectrometer (Thermo Scientific, Wilmington, DE). This laboratory method of DNA extraction is compatible and comparable to a commercial portable handheld cell lysis and DNA extraction kit from Zymo Research (Xpedition sample processor). All species and strains used in this study are summarized in Tables S1 and S2 (Supplemental Information).

### Primer/fluorescent probe design.

We developed unique probe-primer sets for the detection of E. faecium, S. aureus, K. pneumoniae, A. baumannii, and P. aeruginosa. We used an *in silico* bioinformatics approach that analyzes whole-genome sequences and performs comparative searches against all known genomes. The largest usable set of complete genomes was treated as input for each organism. We calculated a multiple-sequence alignment for all of the inputs to determine high-value conserved regions. Simultaneously, we identified unique DNA regions by searching the input genomes against all known bacterial and viral genomes. The result of this process was a set of DNA regions that were unique to the targets and provided the basis for assays that were both sensitive and specific. We identified oligonucleotide primers and probes that targeted the conserved and unique regions of each genome, downselected candidates using chemical design rules required for RPA reactions, and further downselected by reducing candidates with the potential for self-dimerization. In the absence of software that performs high-throughput RPA-specific probe design in a single step, we performed these downselection steps sequentially. Finally, we studied the location of the 3 RPA probe modifications, fluorescent 6-FAM dye, tetrahydrofuran spacer, and black hole quencher 1 (BHQ-1), and adjusted them according to RPA design guidelines to optimize probes.

### RPA assay optimization using RT-PCR.

We generated eight pairs of primers and probes that spanned regions of the genome that were unique to each species and conserved across all sequenced strains of each of the six target organisms. Each primer-probe set was tested in a 25-μl RPA containing 50 ng of target DNA (American Type Culture Collection, Manassas, VA). We tested each primer-probe set against 2 to 6 strains of target DNA isolated from each bacterial species (Table S1). We performed each reaction in triplicate in the presence of the assay exonuclease to determine the time for detection and then without the exonuclease to separate the RPA product on a 4% gel using gel electrophoresis and determine the size of the DNA product. We used a thermal cycler (CFX Touch; Bio-Rad) to maintain reactions at 38°C for 20 min and collected optical readings every 1 min to determine real-time RPA quantification cycle (*C_q_*) values. The size of DNA products was determined using an E-Gel imager (Thermo Fisher Scientific) to image gels. From this set of assays, we selected two primer-probe combinations for testing on the B-chip using three criteria: (i) the shortest time to detection in RPAs, (ii) the smallest standard deviation between replicates, and (iii) production of the appropriate product size.

### B-chip development.

We constructed self-loading B-chips in poly(dimethylsiloxane) (PDMS) due to the ease with which new designs can be created and cartridges prototyped and tested (22, 23). We designed a pattern of microchambers and microchannels using Adobe Illustrator (Adobe, USA) consisting of an inlet chamber (50-μl volume), 16 assay microchambers (1-μl volume), and a reservoir chamber to collect excess sample (30-μl volume) (Fig. 1). A serpentine channel (150 μm wide, 50 μm tall, and 45 mm long) connected the inlet chamber and reservoir chamber. The serpentine channel was connected to the assay microchambers by short channels (150 μm wide, 50 μm tall, and 1.5 mm long). The layout of the channels and chambers and the number of elements are arbitrary and can be redesigned and reconfigured as needed.

We fabricated the master in SU-8 photoresist on silicon wafers using UV photolithography in a two-step procedure. In the first step, we fabricated microchannels by pouring SU-8 3050 (MicroChem, Newton, MA) onto a clean silicon wafer and spin-coated the photoresist at 500 rpm for 10 s using an acceleration of 100 rpm/s, followed by 4,000 rpm at 30 s using an acceleration of 1,000 rpm/s. We baked the photoresist for 5 min at 65°C, followed by 30 min at 95°C and then cooled it at 65°C for 1 min. We exposed the photoresist to a UV light dose of 150 J/cm^2^ through a mask containing the design of the fluidic elements, baked it at 65°C for 1 min and then 95°C for 5 min, and developed unexposed photoresist in SU-8 developer (MicroChem). This first step created a master with 50-μm-tall microchannels. To add the microchambers to the master and complete the B-chip design (Fig. 1A), we performed a second fabrication step to introduce the microchambers that are 500-μm tall using a second layer of SU-8 2100 (MicroChem) and a second photolithography mask that we aligned to the microchannels. After the second SU8 development step, we silanized the masters overnight using heptafluorosilane (Gelest, Inc., Morrisville, PA) in a vacuum chamber. Using soft lithography (33), we imprinted the fluidic structure in the master into a layer of PDMS (Sylgard 184; Dow Corning). The channels were 50 μm tall and 150 μm wide, and the chambers were 2 mm in diameter and 500 μm tall. The microchambers had a volume of 1 μl. We cleaned embossed PDMS layers in isopropanol and double-distilled water and dried with nitrogen gas prior to assembling the B-chip, as described in the section below.

### RPA assays in the B-chip.

To perform RPAs in the B-chip, we added the isothermal RPA mix (species specific primers, fluorescent exoprobe, and buffer) to the lyophilized enzyme pellet (TwistAmp exo kit; TwistDx Ltd., Cambridge, UK) using the TwistDx protocol. We used final concentrations of 420 nM primers and 120 nM fluorescence exoprobe for each assay, as recommended by the manufacturer. We pipetted 1 μl of the mixture (containing enzyme, primers, and probe) into each microchamber and dried it in a laminar flow hood. As a negative control to analyze the fluorescence baseline of the probe without any amplification, we used a reaction mixture lacking primers. We assembled the B-chip by placing a flat thin piece of PDMS (thickness of 5 mm) on top of the microchambers to enclose them and degassed for 60 min in a vacuum chamber at 0.2 atm (Harrick Plasma, Ithaca, NY) (22, 23).

To perform assays, we removed the B-chip from vacuum and loaded it by placing a 50-μl sample solution containing 0.1 to 100 pg/μl bacterial DNA and 14 mM magnesium acetate (MgOAc) at the inlet. Degas-driven flow filled the channels and microchambers; after the sample was consumed, a plug of air filled the central channel and insulated the chambers to prevent cross-contamination of primers and probes. We incubated B-chips at 37°C for 20 to 30 min. The total amount of time between introducing the sample into the device and insulation of the sample in the chambers was ∼15 min. For lab-based experiments, we imaged fluorescence of the 6-FAM on the assay probe using an ImageQuant LAS 4000 (GE Healthcare Life Sciences) with a 460-nm excitation and 520-nm emission filter set, and quantified images using ImageJ (NIH, Bethesda, MD). We calculated the fluorescence fold increase by comparing the fluorescence intensity ratio of reaction mixtures containing primers to our negative controls lacking primers. Background fluorescence was subtracted prior to calculating the fluorescence fold increase.

### Performing RPAs in microplate wells.

We used microplate assays to assess the performance of RPAs for detecting E. faecium, S. aureus, K. pneumoniae, A. baumannii, and P. aeruginosa. We performed 50-μl volume assays in the wells of a 96-well plate (Costar) using the TwistAmp exo kit described above. Prior to the plate assay, all reagents, except for the template DNA and magnesium acetate, were prepared in a master mix and aliquoted into tubes containing an RPA enzyme pellet, DNA was added to each tube, and the mixture in each tube was transferred to a microplate well. The concentrations of primers and fluorescence exoprobe in each assay were 420 nM and 120 nM, respectively. Finally, we added MgOAc to each well (final concentration, 14 mM) to initiate amplification and measured the assay kinetics on a microplate reader incubating at 39°C (Infinite M1000 Pro; Tecan, Switzerland). To detect amplification, we measured 6-FAM fluorescence in 5-min intervals for 1 h using filters compatible with excitation at λ of 488 nm and emission at λ of 520 nm. We determined the fluorescence fold increase by comparing the fluorescence intensity ratio of reaction mixtures containing DNA to a no-template control. Background fluorescence was subtracted prior to calculating the fluorescence fold increase.

### Development and characterization of the portable electronic reader.

We fabricated the body of the portable B-chip detection system from polylactic acid (PLA) plastic using a LulzBot 3D printer (LulzBot, CO, USA). Each device component was designed using SolidWorks (Dassault Systèmes S.A., Vélizy, France) and printed individually (Fig. S1). We used an Arduino microcontroller to control the detection system, which contains Arduino-compatible electronic components. XLamp XB-D Blue LEDs (465 to 485 nm, dominant wavelength range) (Cree, Inc., NC, USA) mounted on DFN6 to DIP10 SMT adapters (Proto-Advantage, Hamilton, Canada) were used to excite 6-FAM, and an OV5642 Arduino-compatible CMOS camera was used to measure fluorescence from the B-chip. We controlled the CMOS using an Arduino shield (Arducam).

The principal supporting body/structure of the device contained a chamber for the B-chip. The chamber has a pin-and-barrel-hinge opening mechanism that enables the chamber to rotate open. The top of the capture chamber forms a base for the OV5642 Arducam-compatible CMOS camera. We placed a manual focus wheel within the detection unit that fits tightly against the CMOS camera focus wheel and makes it possible for the user to manually focus the camera on the B-chip surface, thereby providing us with flexibility to test prototype cartridges that have a range of physical dimensions. An emission filter base is positioned in front of and in very close proximity to the CMOS camera, passes 6-FAM fluorescence, and filters the excitation light from the LEDs.

The bottom of the chamber contains an excitation base that positions excitation filters in front of each LED, a heating bed that incorporates a Peltier heater/cooler (SparkFun Electronics, CO, USA) to keep the B-chip temperature constant, and a body base component that integrates all these components, including a barrel for the hinge mechanism on the main chamber. The B-chip sits in a plastic compartment positioned on top of the Peltier plate and is heated by conduction. A One Wire DS10B20 digital temperature sensor (Maxim Integrated, CA, USA) is in direct contact with the heater to measure and control the B-chip temperature. A battery pack consisting of six 3.6-V, 750-mAh LiFePO4 rechargeable batteries is attached to the back of the capture chamber. Two parallel sets of four batteries in series provide continuous 12-V power supply. A Seeed Motor Shield (Seeed Technology Co., Shenzhen, China) regulates power supply to the Arduino.

### Statistical analysis.

All data are presented as mean values of the results from at least 3 independent experiments and include values for the standard error of the mean.

## Supplementary Material

Supplemental material
