# Supplementary material for: Detection of ESKAPE Bacterial Pathogens at the Point of Care Using Isothermal DNA-Based Assays in a Portable Degas-Actuated Microfluidic Diagnostic Assay Platform
Source: Appl Environ Microbiol. 2017 Feb 1;83(4):e02449-16. doi: 10.1128/AEM.02449-16 (PMC5288812; doi:10.1128/AEM.02449-16)
Supplement: Supplemental material [file supp_83_4_e02449-16__index.html]

Supplemental material 

# Detection of ESKAPE Bacterial Pathogens at the Point of Care Using Isothermal DNA-Based Assays in a Portable Degas-Actuated Microfluidic Diagnostic Assay Platform

## Supplemental material

- Supplemental file 1 -

  CAD cross-section of the B-chip reader (Fig. S1); selection of primer and probe combinations (Fig. S2); examination of fluorescence intensity (Fig. S3); quantification of DNA concentration (Fig. S4); specificity assay (Fig. S5); testing the specificity towards different related strains (Fig. S6); testing RPA after lysis of PAO1 (Fig. S7); fluorescence standard curves of B-chip in ImageQuant and B-chip reader (Fig. S8); DNA samples used in this study (Table S1); bacterial species and strains used in this study (Table S2); signature sequences of the target loci, forward/reverse primers, and probe sequences for the ESKAPE collection for the assay design used in this study (Table S3).

  PDF, 3.4M
