# Supplementary material for: Detection of ESKAPE Bacterial Pathogens at the Point of Care Using Isothermal DNA-Based Assays in a Portable Degas-Actuated Microfluidic Diagnostic Assay Platform
Source: Appl Environ Microbiol. 2017 Feb 1;83(4):e02449-16. doi: 10.1128/AEM.02449-16 (PMC5288812; doi:10.1128/AEM.02449-16)
Supplement: Supplemental material [file AEM.02449-16_zam999117657s1.pdf]

Supplemental Information:

Detection of ESKAPE bacterial pathogens at the point-of-care using isothermal DNA-based assays in a portable, de-gas microfluidic diagnostic assay platform

[#]Lars D. Renner[1†,2], [#]Jindong Zan[1†], Linda I. Hu[1†], Manuel Martinez[3], Pedro J. Resto[1†,3], Adam C. Siegel[1†], Clint Torres[4∫], Sara B. Hall[4Π], Tom R. Slezak[4√], Tuan H. Nguyen[4√]*, Douglas B. Weibel[1†‡§]*

1. *Department of Biochemistry†, Department of Biomedical Engineering‡, Department of Chemistry§, University of Wisconsin-Madison, Madison, WI 53706*

2. *Leibniz Institute of Polymer Research, Dresden, Germany, 01069*

3. *Department of Mechanical Engineering University of Puerto Rico at Mayagüez, Mayagüez, PR 00680.*

4. *Computations Directorate∫, Physical and Life Sciences DirectorateΠ, Global Security Principal Directorate√, Lawrence Livermore National Laboratory, Livermore, CA 94551*

#These authors contributed equally to this work

*Author to whom correspondence should be addressed:
Douglas B. Weibel
University of Wisconsin-Madison
440 Henry Mall
Madison, WI 53706
Phone: (608) 890-1342
E-mail: weibel@biochem.wisc.edu

Tuan H. Nguyen
Global Security Principal Directorate
Lawrence Livermore National Laboratory
7000 East Avenue L-170
Livermore, CA 94551
Phone: (925) 422-2516
E-mail: thn@llnl.gov

Fig. S1. CAD cross-section of the 'B-chip' reader. CAD cross-section of the reader housing with labeled legend of the main parts.

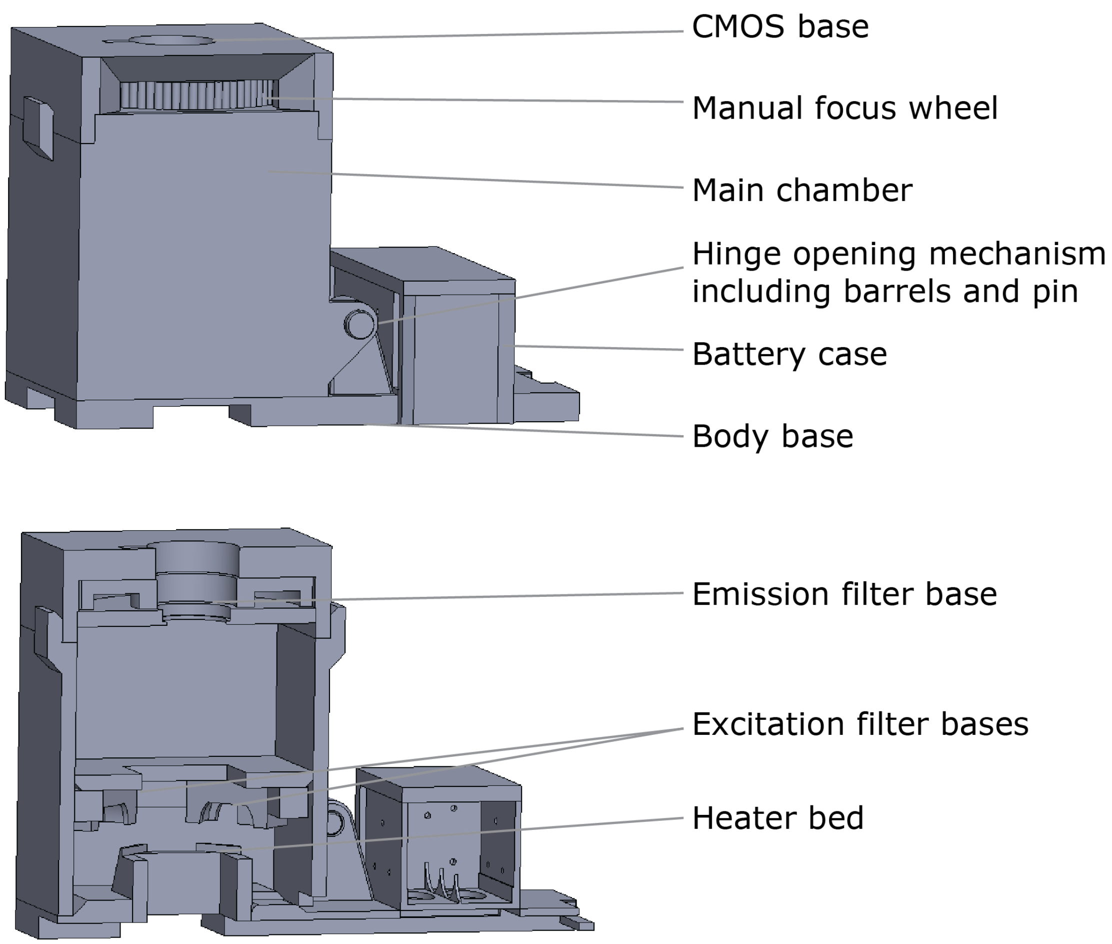

Fig. S2. Selection of primer and probe combinations. Examples images of fluorescence of RPA assays for tested strains with or without primers for up to two primer-probe combinations per strain in the microchambers for a single pathogenic strain, primer pair and DNA sample (+ indicates sample DNA with primers, - indicates sample DNA without primers).

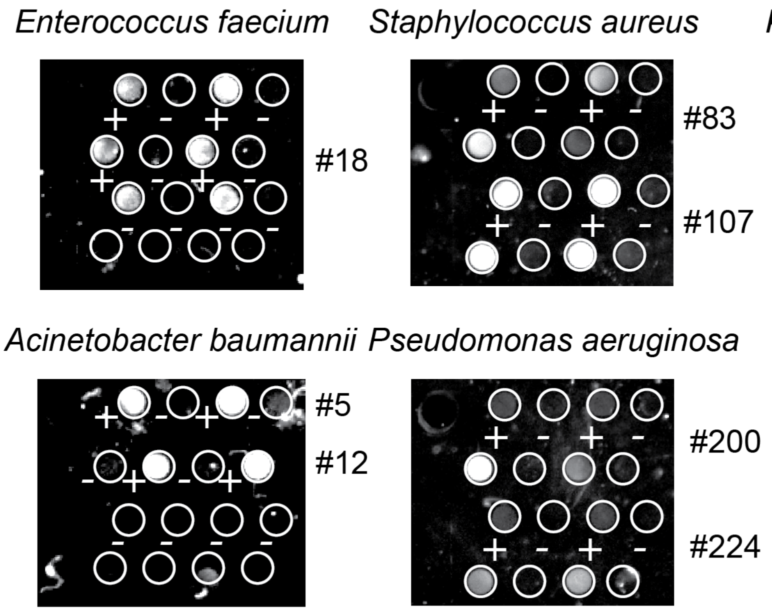

Fig. S3. Examination of fluorescence intensity. 6-FAM standard curve comparison between plate reader and B-chip (ImageQuant) to assess the performance of 1μL vs. 50μL total reaction volume (n=3).

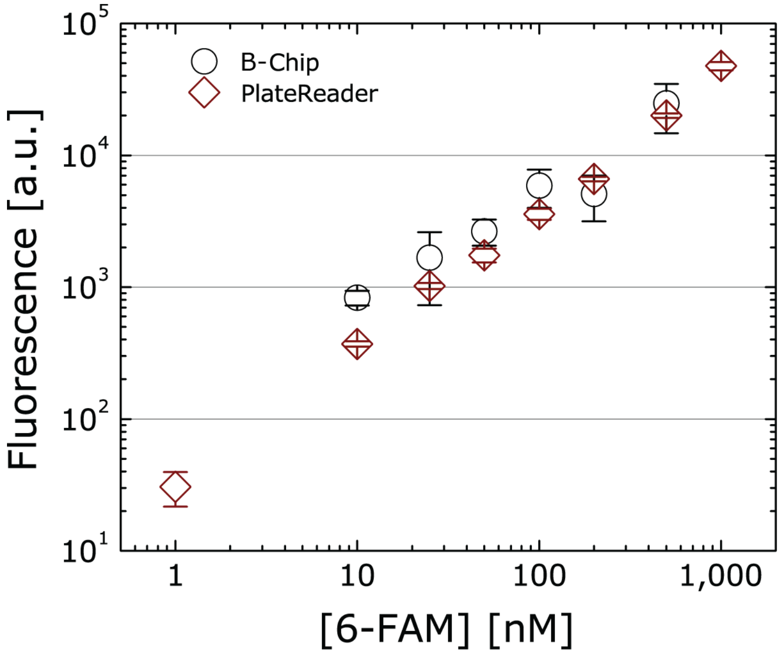

Fig. S4. Quantification of DNA concentration. Measurement of DNA concentration (A) on 'B-chip' and (B) with plate reader by extrapolating the fluorescence fold increase with a linear fit on a double log plot (n=3). *A. baumannii* (Ab), *E. faecium* (Ef), *K. pneumoniae* (Kp), *P. aeruginosa* (Pa) and *S. aureus* (Sa).

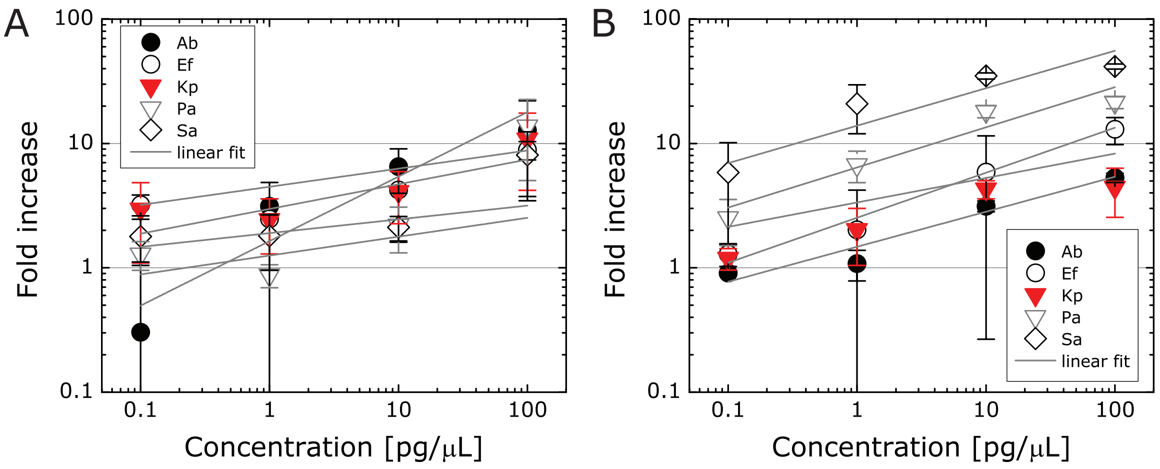

Fig. S5. Specificity assay: results of plate reader assays analyzing the specificity of the unique primer set against the different pathogenic DNA from the ESKAP(e) collection (n=2). Every primer and probe set is unique and specific to its DNA. The red line indicates no amplification.

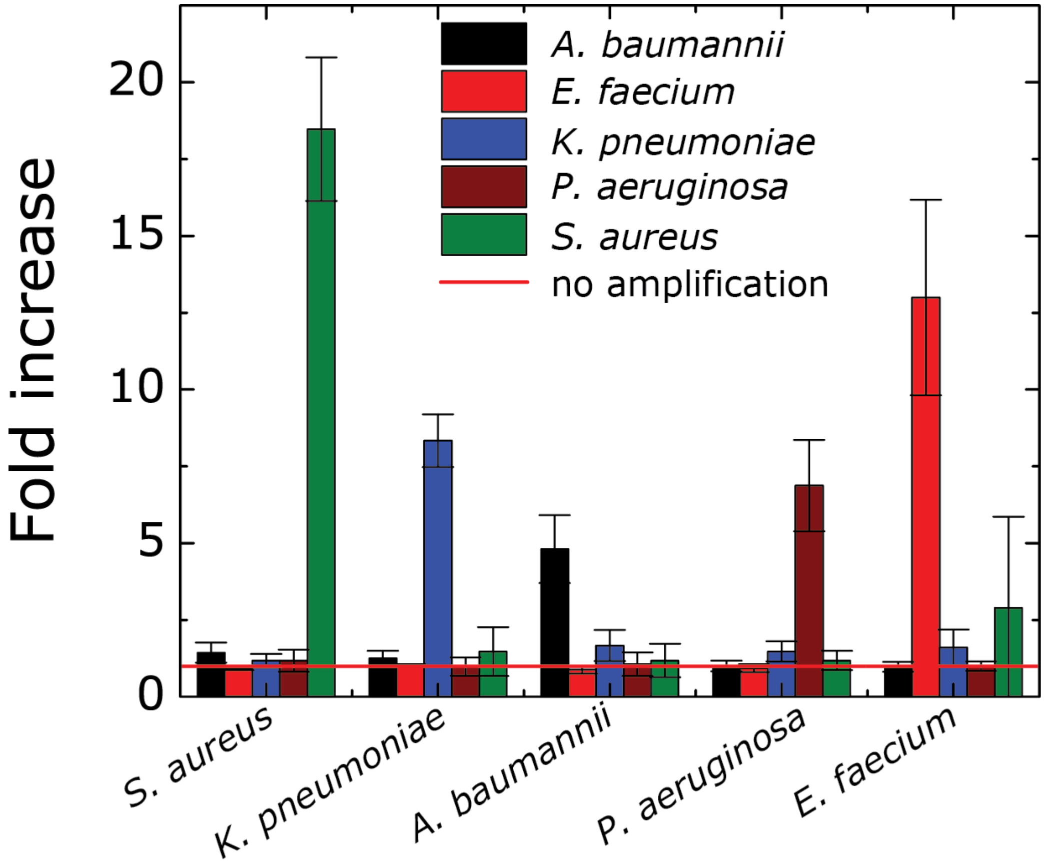

Fig. S6. Testing the specificity towards different related strains. Examples of on chip fluorescence data for the analysis of specificity of ESKAP(e) species towards closely related strains of *Pseudomonas (Ps) sp. and Staphylococcus (St.) sp*. Red dashed box indicates primer and probe, white dashed box indicates probe only (no primer).

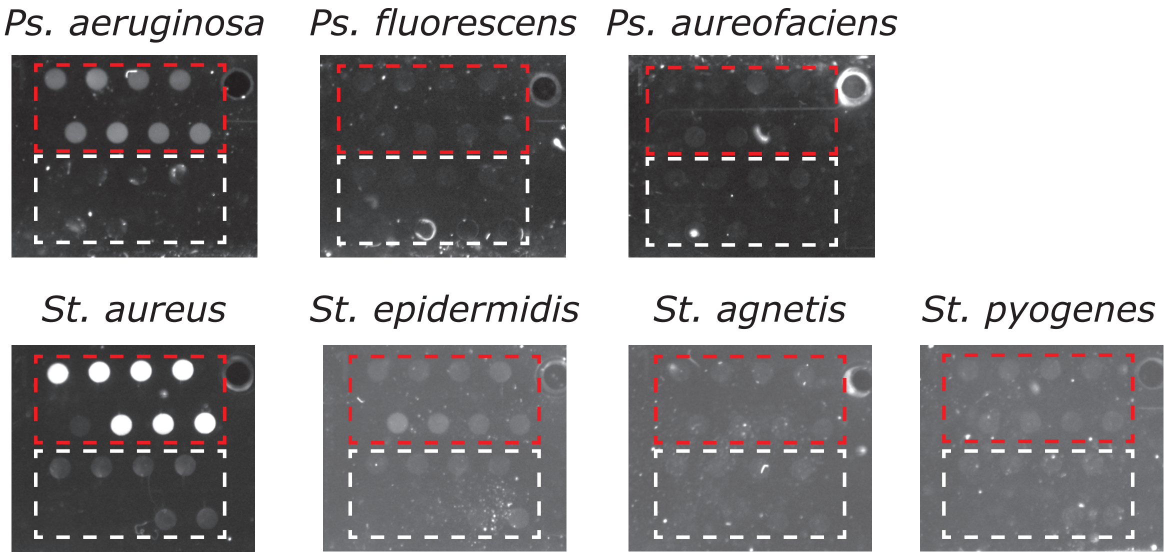

Fig. S7. Testing RPA after lysis of PAO1. (A) Examples of on chip fluorescence data for the analysis of different dilutions of freshly lysed *Pseudomonas aeruginosa* PAO1 samples in comparison to pure *P. aeruginosa* DNA (from ATCC, compare Table S1). Green circles indicate primer and probe, red circles indicate probe only (no primer). (B) A plot depicting the fold increase of fluorescence levels (ratio) between primer and no primer of the different dilutions and pure *P. aeruginosa* DNA (n=3).

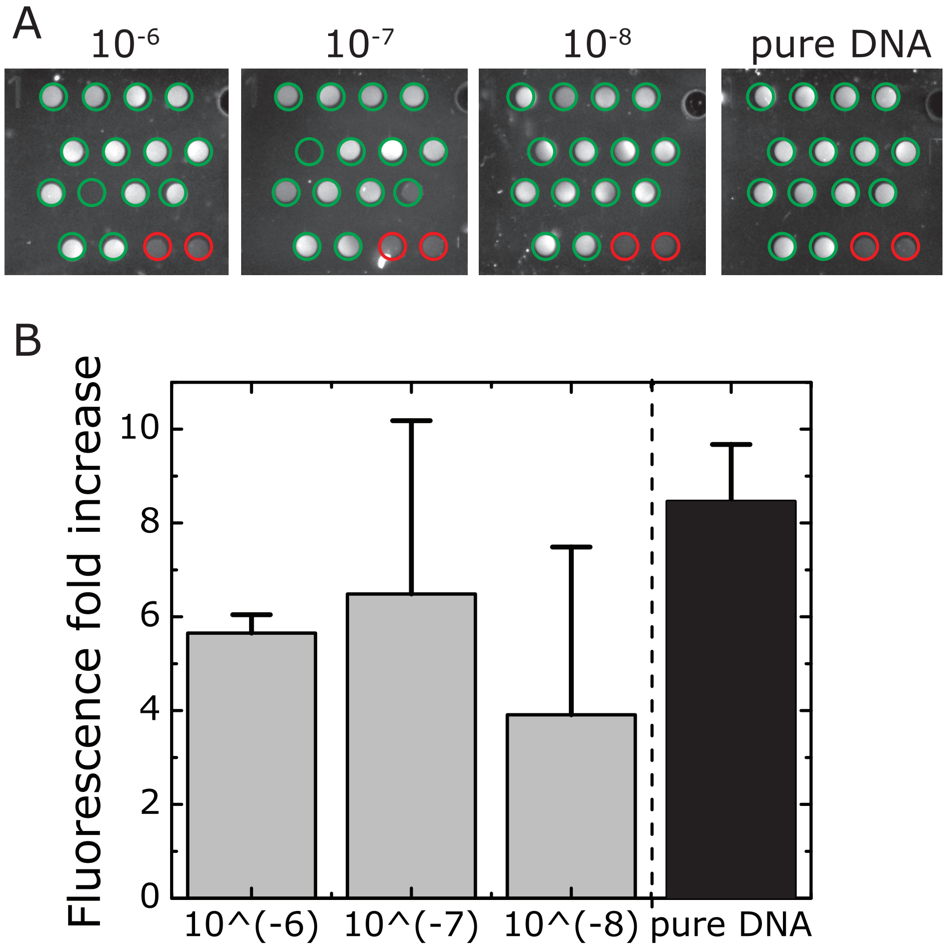

Fig. S8. Fluorescence standard curves of B-Chip in Image Quant and B-Chip Reader. (A) Comparison of fluorescent readouts of 6-FAM samples between 50-10,000nM with ImageQuant and B-Chip Reader. Corresponding standard curves for 2 independent measurements from (B) ImageQuant and (C) B-Chip Reader. Microchambers are 500 μm tall.

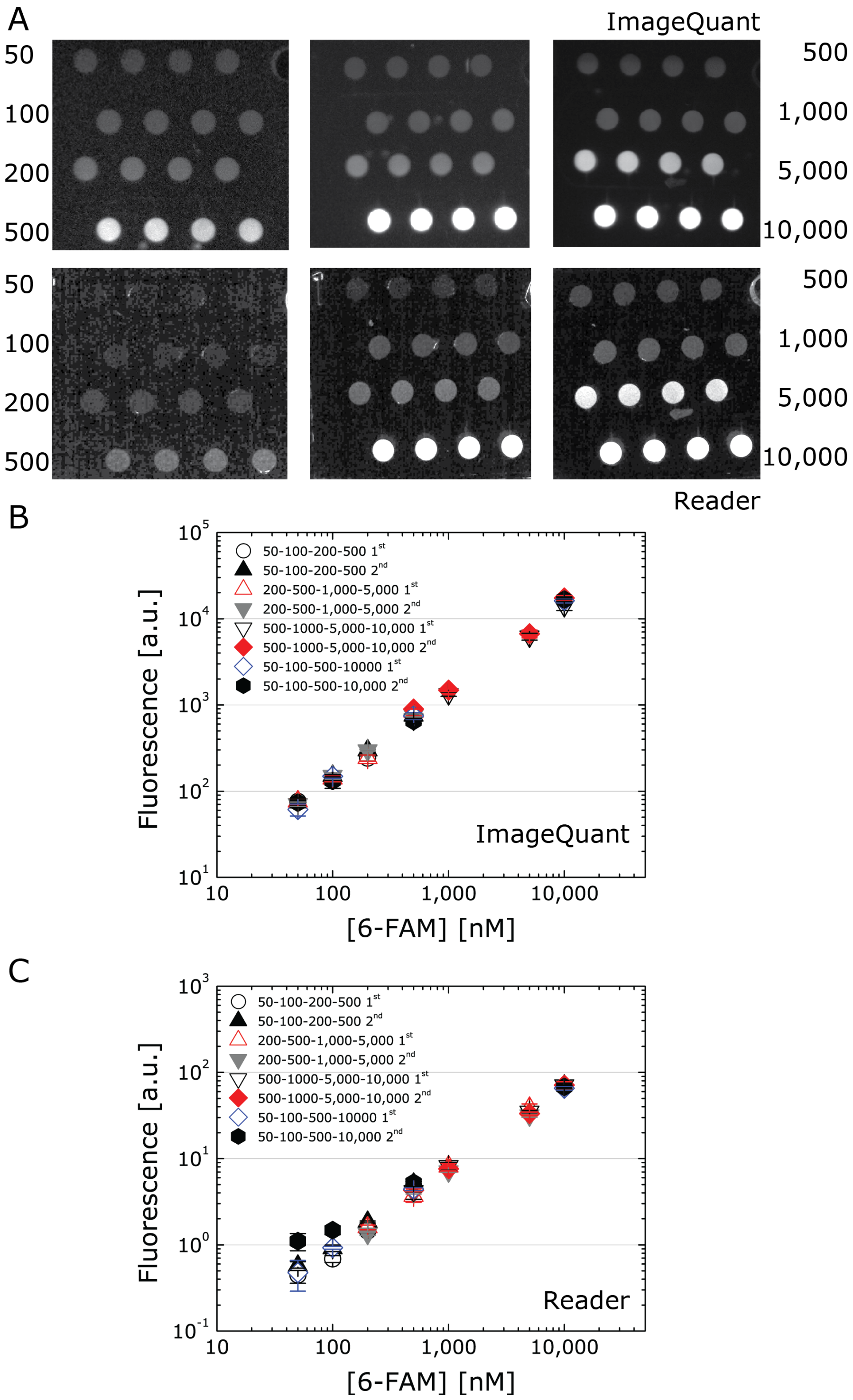

A
ImageQuant
50
100
200
500
500
1,000
5,000
10,000
Reader
B
50-100-200-500 1st
50-100-200-500 2nd
200-500-1,000-5,000 1st
200-500-1,000-5,000 2nd
500-1000-5,000-10,000 1st
500-1000-5,000-10,000 2nd
50-100-500-10000 1st
50-100-500-10,000 2nd
ImageQuant
Fluorescence [a.u.]
[6-FAM] [nM]
10
100
1,000
10,000
C
Reader
Fluorescence [a.u.]
[6-FAM] [nM]

Table S1. DNA samples used in this study.

**Species name**	**DNA ATCC Number**
Staphylococcus aureus	BAA-1556D-5
Staphylococcus aureus	700699D-5
Staphylococcus aureus	35556D-5
Staphylococcus aureus	10832D-5
Staphylococcus aureus	25923D-5
Staphylococcus aureus	700698D-5
Klebsiella pneumoniae	700721D-5
Klebsiella pneumoniae	700603D-5
Klebsiella pneumoniae	BAA-1706D-5
Klebsiella pneumoniae	BAA-1246D-5
Klebsiella pneumoniae	BAA-1705D-5
Acinetobacter baumannii	17978D-5
Acinetobacter baumannii	BAA-1710D-5
Acinetobacter baumannii	19606D-5
Acinetobacter baumannii	BAA-1605D-5
Pseudomonas aeruginosa	17933D
Pseudomonas aeruginosa	47085D-5
Pseudomonas aeruginosa	9027D-5
Pseudomonas aeruginosa	27853D-5
Pseudomonas aeruginosa	15442D-5
Enterococcus faecium	51559D-5
Enterococcus faecium	BAA-472D-5

Table S2. Bacterial species and strains used in this study.

**Bacterial species**	**Source**
Acinetobacter baumannii	Clinical isolate from UW-Madison, Welch lab (Medical Microbiology and Immunology)
Klebsiella pneumonia ATCC BAA-1706	ATCC
Staphylococcus aureus	Clinical isolate from UW-Madison, Welch lab (Medical Microbiology and Immunology)
S. epidermidis	Clinical isolate from UW-Madison, Welch lab (Medical Microbiology and Immunology)
S. agnetis	Clinical isolate from UW-Madison, Welch lab (Medical Microbiology and Immunology)
S. pyogenes	Clinical isolate from UW-Madison, Welch lab (Medical Microbiology and Immunology)
Pseudomonas aeruginosa PAO1	Strain from Dr. Pete Greenberg (University of Washington, Seattle) reference: (1)
P. aureofaciens 30-84	Strain from Dr. Michael G. Thomas (University of Wisconsin-Madison) reference: (2)
P. fluorescens WCS365	Strain from Dr. Michael Thomas (UW-Madison) reference: (3)
Enterococcus faecium	Clinical isolate from UW-Madison, Welch lab (Medical Microbiology and Immunology)

Table S3. Signature sequences of the target loci, forward/reverse primers and probe sequences for the ESKAPE collection for the assay design used in this study.

ESKAPE Signature Bioinformatics

Target Sequence	Forward Primer ID	Forward Primer Sequen	Reverse Primer ID	Reverse Primer Sequence	Probe ID	Probe Sequence	Amplicon Start Location	Amplicon End Location	Amplicon Length [bp]	AmpliconSeq
gb|CP003846.1|gi|522324570|Acinetobacter baumannii BJAB07104, complete genome[sequence_id 834583][seq_data_id 8343543][tax_node_id 1096995]	ab_05F	TGCTTGTGGAAGTGGATCTGTAATATTGGTGGT	ab_05R	TGGGCTACTTGTTGCGACAGGCATTTATCTAAT	ab_05P	GCGCGTACCTTCCCTGTTGTGTTGGGCTTAGCAGTGCTGGGCTATGCGG	429756	429939	184	TGCTTGTGGAAGTGGATCTGTAATATTGGTGGTTTCGGTTAGGATGGCTGGTGAGCTGACTTGCAAACGACCCATCGCAAACAAAAATAAGTTAACCGCATAGCCCAGCACTGCTAAGCCCAACACAACAGGGAAGGTACGCGCACGTAAAATTAGATAAATGCCTGTCGCAACAAGTAGCCCA
gb|CP003846.1|gi|522324570|Acinetobacter baumannii BJAB07104, complete genome[sequence_id 834583][seq_data_id 8343543][tax_node_id 1096995]	ab_12F	TCTTCTCTCATACGATGAACGAAGCAAGAGCAG	ab_12R	ATCAAGAATACGGTTAGATGTGTTCGCATCCAG	ab_12P	CGATCCAGCCAACGCAAGAGCAAGCCTTGGTGGCTCGTCAACTGGCAAC	599665	599840	176	TCTTCTCTCATACGATGAACGAAGCAAGAGCAGCAACCAATACTGCTGCTGTTTCTCAATCGATCCAGCCAACGCAAGAGCAAGCCTTGGTGGCTCGTCAACTGGCAACTTTAGTAGACCGCCAACATTATTTAAATATGCGTCTGGATGCGAACACATCTAACCGTATTCTTGAT
gb|CP006620.1|gi|529202214|Enterococcus faecium Aus0085, complete genome[sequence_id 836140][seq_data_id 8385185][tax_node_id 1305849]	ef_57F	TCAAACTATCCGACTTGCTTCCAGTTATGCCTT	ef_57R	AATTGCCAAGCAGCTCCTTGTTGACCTGTATAT	ef_57P	GGAAGCGGGTGTAGCGATGGTGGTCTAGGATTACTCCAATCCTTGGGTG	613246	613542	297	TCAAACTATCCGACTTGCTTCCAGTTATGCCTTAGGAGAACTTATTAAAGATGCACTGCTTCAAGAAGTAGAACAAATTATTGTCACTTTAGGAGGAAGCGGGTGTAGCGATGGTGGTCTAGGATTACTCCAATCCTTGGGTGCTTCTTTAGAAGGGTACACAGAAGGAAATCCTTTATTATCTACTAAAAAACTTCATTTTGATCGAAGAAACGTTCTTGTTTCTAAAGTAAAATTGATTATCGCTGCAGATGTTACCAATCCATATACAGGTCAACAAGGAGCTGCTTGGCAATT
gb|CP006620.1|gi|529202214|Enterococcus faecium Aus0085, complete genome[sequence_id 836140][seq_data_id 8385185][tax_node_id 1305849]	ef_62F	TACTCGTAGGAACAGCAATGTTGATGCTCTCCT	ef_62R	ATCAACAATGTTGCAAAAGCTACGTTGCCTAAC	ef_62P	GGCCGTTTCGCTGGTTTTATCAGCGGTTGGGGTTACTGGCTCACATGCT	621441	621630	190	TACTCGTAGGAACAGCAATGTTGATGCTCTCCTTGTCGATCAATAATATCAATAAAAAACGTCCAGATTTAAAAGGCGGGATCTTTAGTTACGCAGAAGAAGGTTTTGGCCGTTTCGCTGGTTTTATCAGCGGTTGGGGTTACTGGCTCACATGCTGGTTAGGCAACGTAGCTTTTGCAACATTGTTGAT
gnl|PRJNA177782|organism=Klebsiella_pn>|gb|CP006648.1|gi|549815675|Klebsiella pneumoniae CG43, complete genome[sequence_id 842451][seq_data_id 8599854][tax_node_id 1244085]	kp_07F	CCGCGACCACTTTCTCAATGGCCGGCTGCACGGCCTG	kp_07R	AACAGTGAGTCCACGCTTTACCAGAAGATGCTC	kp_07P	CCGACACCGATGCTTCGCGTCTGTTCAGCACTGATGAGGTGGTGCCGGG	2388833	2389025	193	CCGCGACCACTTTCTCAATGGCCGGCTGTACGGCCTGCTCCCAGGCCTGACGGGTAAACATTCCCGGCACCACCTCATCAGTGCTGAACAGACGCGAAGCATCGGTGTCGGCAGTCATATCCGCCAGGCGCATATCGGCATACTGATTCGCCACCTGCGCGAGCATCTTCTGGTAAAGCGTGGACTCACTGTT
gnl|PRJNA177782|organism=Klebsiella_pn>|gb|CP006648.1|gi|549815675|Klebsiella pneumoniae CG43, complete genome[sequence_id 842451][seq_data_id 8599854][tax_node_id 1244085]	kp_10F	GTAATAAGCGTCTGGTGAGCCAGAAAACGCTC	kp_10R	TCACTTACGGTGAGGTATACCACTATAAGCTTCG	kp_10P	AGTTGGCGGTCTCCGTCTGCGGGTCGATTTTATCGCCACGCTCCCGGTG	2398713	2398848	136	GTAATAAGCGTCTGGTGAGCCAGAAAACGCTCATGGTCGAGGCGGGCATAGAAGTTGGCGGTCTCCGTCTGCGGGTCGATTTTATCGCCACGCTCCCGGTGGCGAAGCTTATAGTGGTATACCTCACCGTAAGTGA
gi|106896550|gb|AAQW01000001.1| Pseudomonas aeruginosa PACS2 chromosome, whole genome shotgun sequence|Pseudomonas aeruginosa PACS2 Pseudomonas aeruginosa PACS2, whole genome shotgun sequencing project from NCBI on Nov 08 2013 12:18PM[sequence_id 844736][seq_data_id 8907668][tax_node_id 388272]	pa_200F	CATCAGGCTCTTGATCAGCAACGGATACTGATA	pa_200R	GCAAATCACTCTTTTGACTGATGAACAAAGCCC	pa_200P	CCGACGGCTCTAGAGTCGGCCTCATCCTGACAACGAACCCGCTCCCAGG	5084276	5084440	165	CATCAGGCTCTTGATCAGCAACGGATACTGATAAGCGCCGGCGGCGGCCGGGATGAGACGGGTCTTCAGCATGGGGCGTACACCTGGGAGCGGGTTCGTTGTCAGGATGAGGCCGACTCTAGAGCCGTCGGCGGGCTTTGTTCATCAGTCAAAAGAGTGATTTGC
gi|106896550|gb|AAQW01000001.1| Pseudomonas aeruginosa PACS2 chromosome, whole genome shotgun sequence|Pseudomonas aeruginosa PACS2 Pseudomonas aeruginosa PACS2, whole genome shotgun sequencing project from NCBI on Nov 08 2013 12:18PM[sequence_id 844736][seq_data_id 8907668][tax_node_id 388272]	pa_224F	CAATATGGAGACCGTTTTCAGGTATTTCAAGGC	pa_224R	TGACTTGCATGCAGGATTACCGTCTATTAGAGC	pa_224P	CGCGGCAGATTGTCACCTGCTAGGCTGTCCATCCAGTGTAGATGGCTCC	5757469	5757706	238	CAATATGGAGACCGTTTTCAGGTATTTCAAGGCACGCGGCAGATTGTCACCTGCTAGGCTGTCCATCCAGTGTAGATGGCTCCAGCTTCGCAGCCAGGTGAACTGGCGCTTGGCCAACTGGCGGGTAGCGATGATGCCGCGTTCGGTCATTTCAGCGTAGGACAGCTTGCCATCCAGGTAATCCCACACCTGACGGTAACCCACGGCTCTAATAGACGGTAATCCTGCATGCAAGTCA
gb|CP003603.1|gi|545580417|Staphylococcus aureus subsp. aureus SA957, complete genome[sequence_id 841766][seq_data_id 8598024][tax_node_id 1201010]	sa_083F	CACGACATCATTGATAAATCGCTTGAAATGTTG	sa_083R	TCACCTGGGATATCAAAGTCCGTTACATGTTGT	sa_083P	CATAATACCTGCGCCATCACCAGTGATGCCATTTGCGCCGACCCCGCCC	479644	479801	158	CACGACATCATTGATAAATCGCTTGAAATGTTGCGACGTTTAGATCACAGGGGCGGGGTCGGCGCAGATGGCATCACTGGTGATGGCGCAGGTATTATGACTGAAATACCTTTTGCATTTTTCAAACAACATGTAACGGACTTTGAAATCCCAGGTGA
gb|CP003603.1|gi|545580417|Staphylococcus aureus subsp. aureus SA957, complete genome[sequence_id 841766][seq_data_id 8598024][tax_node_id 1201010]	sa_107F	CCAATGGCAATCAATTGGGATCTTAGTTGTTTT	sa_107R	AACATTTGTCAAACTTGCAAATGCAAAACCTGT	sa_107P	GAGGAGCAGCCAATGGTATCGCTTGGGTTGTTCAGTCAGCGCATGCTGG	686812	686983	172	CCAATGGCAATCAATTGGGATCTTAGTTGTTTTAAACCTGTTTTTAGCATGGTTCTTTATTTATTTTGATTGGGGTCAAAAAGCAGTAAGAGGAGCAGCCAATGGTATCGCTTGGGTAGTTCAGTCAGCGCATGCTGGTACAGGTTTTGCATTTGCAAGTTTGATAAATGTT

References:

1. **Whiteley, M, Lee, KL, Greenberg, EP**. 1999. Identification of genes controlled by quorum sensing in *Pseudomonas aeruginosa*. Proceeding of the National Academy of Sciences **96**: 13904-13909.
2. **Delaney, SM, Mavrodi, DV, Bonsall, RF, Thomashow, LS.** 2001. PhzO, a gene for biosynthesis of 2-hydroxylated phenazine compounds in *Pseudomonas aureofaciens*. Journal of Bacteriology **183:** 318-327.
3. **O'Toole, GA, Kolter, R**. 1998. Initiation of biofilm formation in *Pseudomonas fluorescens* WCS365 proceeds via multiple, convergent signalling pathways: a genetic analysis. Molecular Microbiology **28**: 449-461.
